# Multiyear trend in reproduction underpins interannual variation in gametogenic development of an Antarctic urchin

**DOI:** 10.1038/s41598-021-98444-4

**Published:** 2021-09-22

**Authors:** Rebecca De Leij, Lloyd S. Peck, Laura J. Grange

**Affiliations:** 1 University of Southampton, Waterfront Campus, European Way, Southampton, SO14 3ZH UK; 2 British Antarctic Survey, High Cross, Madingley Rd, Cambridge, CB3 0ET UK; 3 School of Ocean Sciences, Bangor University, Bangor, LL57 2DG Gwynedd, North Wales UK

**Keywords:** Environmental sciences, Ecology, Climate-change ecology

## Abstract

Ecosystems and their biota operate on cyclic rhythms, often entrained by predictable, small-scale changes in their natural environment. Recording and understanding these rhythms can detangle the effect of human induced shifts in the climate state from natural fluctuations. In this study, we assess long-term patterns of reproductive investment in the Antarctic sea urchin, *Sterechinus neumayeri*, in relation to changes in the environment to identify drivers of reproductive processes. Polar marine biota are sensitive to small changes in their environment and so serve as a barometer whose responses likely mirror effects that will be seen on a wider global scale in future climate change scenarios. Our results indicate that seasonal reproductive periodicity in the urchin is underpinned by a multiyear trend in reproductive investment beyond and in addition to, the previously reported 18–24 month gametogenic cycle. Our model provides evidence that annual reproductive investment could be regulated by an endogenous rhythm since environmental factors only accounted for a small proportion of the residual variation in gonad index. This research highlights a need for multiyear datasets and the combination of biological time series data with large-scale climate metrics that encapsulate multi-factorial climate state shifts, rather than using single explanatory variables to inform changes in biological processes.

## Introduction

Reproduction is a fundamental process for all life. Reproductive periodicities are often intrinsic rhythms entrained by external cues that aid synchronicity in reproduction, as well as timing the arrival of vulnerable early life-stages with favourable conditions^1,2^. To understand environmental influences on reproductive processes, innate reproductive periodicities must be detangled from environmental fluctuations, both for regular seasonal variation and isolated events.

Seasonal and annual reproductive periodicities have been well documented in marine invertebrates. Evidence suggests that local environmental cues including photoperiod^3,4^, water temperature^5,6^, food availability^7,8^, and lunar cycles^6,7,9^, play roles in regulating gametogenesis. However, reproductive cycles and their drivers still remain challenging to interpret, since they are often regulated by the interplay of multiple climate variables^10^.

Changes to the climate state can affect populations at local, regional and global scales^11–13^. For example, the large-scale climate metric, Southern Oscillation Index (SOI), is most known for its regional impacts on the tropical Pacific^11,14^. However, strong links have been found between El Niño- Southern Oscillation (ENSO) and extreme events such a heat waves and storms, across the globe^15^. SOI and extreme events drive ecological processes^16,17^, and more specifically, reproductive processes^18,19^, with some impacts reaching as far as Antarctica^20,21^. Multi-factorial indices, rather than single variables, instead provide a context for large-scale oceanographic variation, and hence integrate both locally measured components of weather and rarer, infrequent, extreme events^10^.

Although regulation of gametogenesis, reproductive cycles and synchronisation of spawning events are undoubtedly influenced by environmental factors, temporal patterns in some species are largely regulated by endogenous rhythms that govern not only reproduction, but other developmental and biological functions such as growth and seasonal activity^22,23^. These rhythms may be caused by an internal oscillator, allocating energy based on life history requirements on seasonal, annual or decadal cycles^22^.

To understand how climate interacts with fundamental biological processes such as reproduction, we first need to identify these innate internal rhythms^24,25^, especially for slow paced species, including many Antarctic taxa. Antarctic marine invertebrates have adapted in situ over millennia to unique conditions characterised by low and stable temperatures and extreme seasonality in light and food availability^26^. Many species have adapted to control and minimise energy expenditure^2,27^, and exhibit extended reproductive cycles, where gametogenesis often takes 18–24 months to complete instead of the 6–12 months characteristic of their temperate counterparts^28,29^.

The Antarctic sea urchin, *Sterechinus neumayeri,* is one of the most functionally important Antarctic shallow marine species. It has a circumpolar distribution and is the most dominant echinoid in the near-shore benthic community, having recorded abundances up to 223 individuals m^−2^^30^. *Sterechinus neumayeri* is an important predator and grazer. It is also a model research species due to its ease of husbandry, including laboratory spawning and larval culture^31,32^. The urchin also has an extended 18–24 month gametogenic cycle^28^.

**Figure 1:**
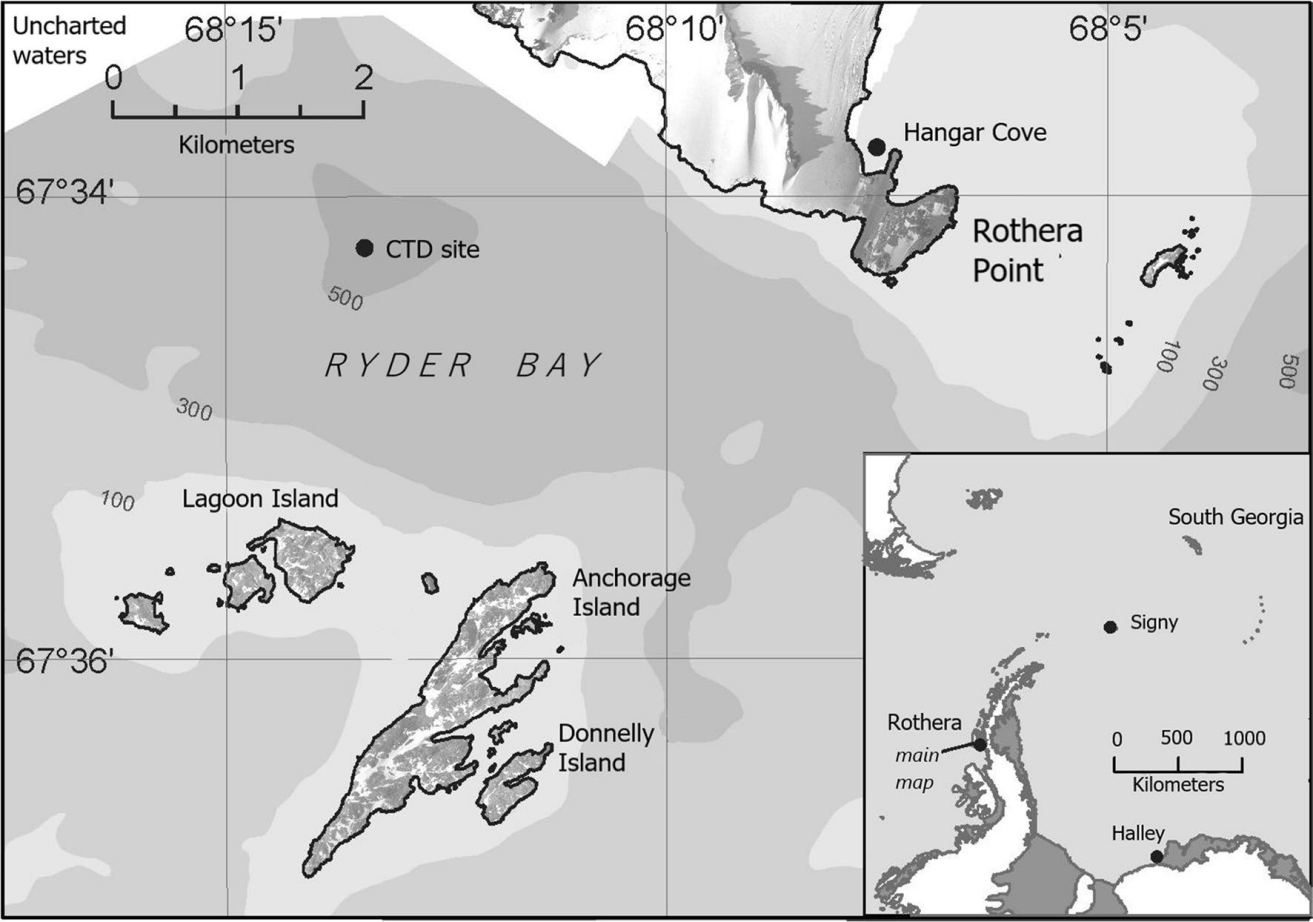
Location of Hangar Cove study site at Rothera Point, Adelaide Island, Antarctica (67°33′54.2"S 68°07′13.1"W) and insert map showing Rothera Point on Western Antarctic Peninsula. Marine environmental data are collected at the site south west of Rothera Point as part of the British Antarctic Survey Rothera Time Series monitoring programme (RaTS). Large-scale map indicates the position of Rothera Research Station on Adelaide Island, on the Western Antarctic Peninsula. Figure modified from Grange et al. (2011).

This study aims to document the reproductive ecology of *S. neumayeri* across multiple years, characterising seasonal and interannual variability and the key factors underpinning reproductive allocation. To this purpose, we investigated the reproductive cycle of *S. neumayeri* over a seven-year period (2012–2018). Seasonal and interannual variations in reproductive condition were explored in relation to locally measured environmental variables (e.g., temperature, chlorophyll a, etc.) and the regional climate metrics SOI and SAM due to their influence in the Southern Ocean and connection with extreme events (Fig. 1).

## Results

The total number of female urchins collected each year exceeded the number of males. Overall, the sex ratio was significantly skewed towards females at 1.32:1 (chi-squared = 13.38, p < 0.001, n = 718) (Supplementary information, Table S1). The gonad index was significantly higher in females (t(661) = 2.26 , p = 0.024) however there was no significant difference in animal size between the sexes (Supplementary information, Table S2).

### Seasonal cycles

**Figure 2:**
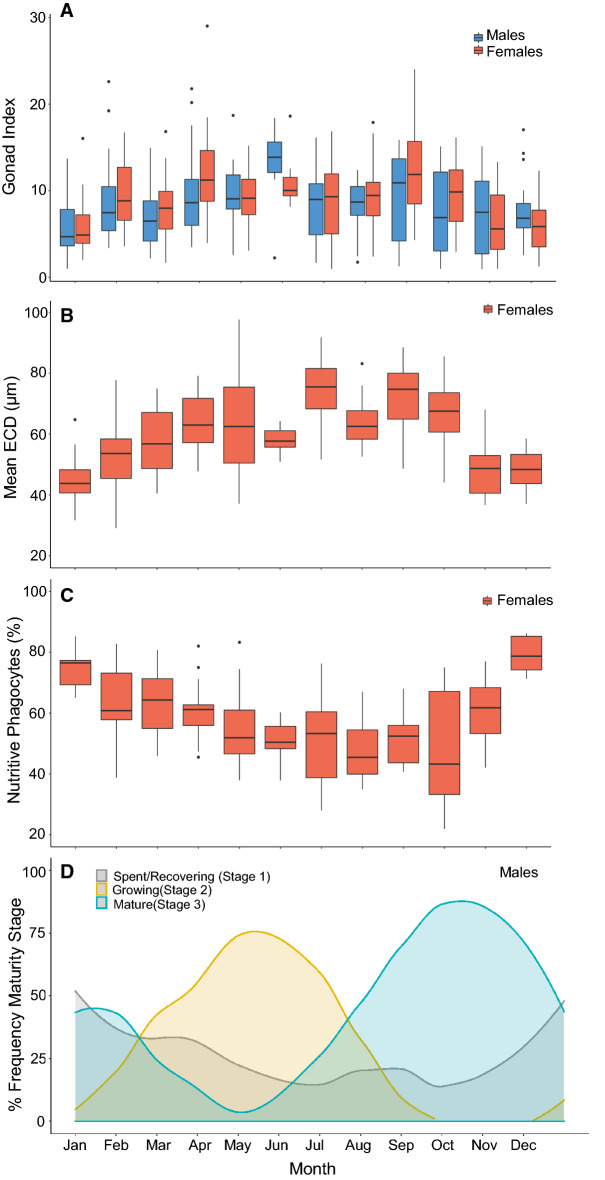
Monthly changes for *Sterechinus neumayeri* in (**A**) gonad index for males (blue) and females (red); (**B**) mean equivalent circular diameter (ECD) of oocytes present in female gonads; (**C**) percentage (%) of gonad tissue in females composed of nutritive phagocytes; (**D**) percentage frequency (%) of male gonad maturity stages where frequencies are smoothed by the function y ~ x using the local regression smoother (LOESS) method. The smoothing span was chosen to reflect seasonal changes. Data as box plots are displayed with the central line in the boxes representing the median value, the upper and lower hinges representing the 25th and 75th percentiles, and the upper/lower whiskers representing the largest/smallest value, no further than 1.5 times the interquartile range from the hinge. All data outside these ranges are plotted as points.

**Figure 3:**
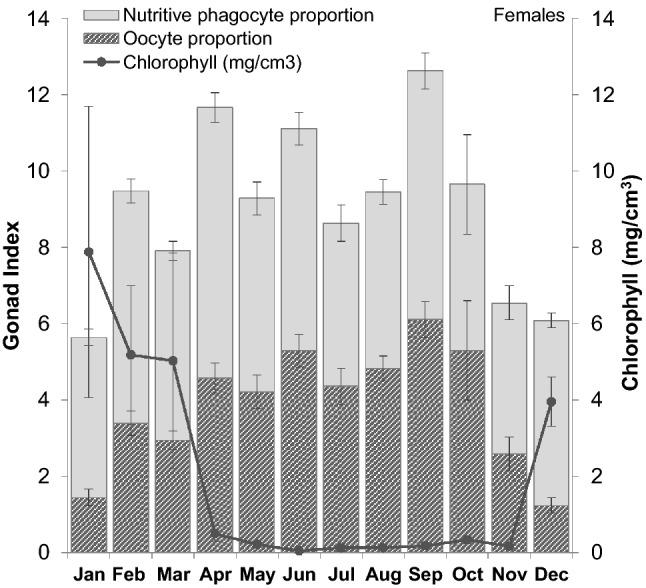
Monthly female gonad index as proportional area of nutritive phagocytes (NP) and oocytes. Monthly data for proportions are ± the standard error of the NP or oocyte equivalent GI based on replicate months. Chlorophyll data are represented on the secondary y-axis and have been averaged from the time series (2012–2018), ± standard error.

Males and females exhibited seasonal cycles in gonad index (GI), oocyte size, nutritive phagocyte proportions and male maturity (Fig. 2), where both males and females presented synchronous cycles, although there was considerable individual variability. GI peaked in June and September for both males (12.8 ± 1.97 S.E. and 9.54 ± 1.48 S.E., respectively) and females (11.1 ± 1.05 S.E. and 12.6 ± 1.24 S.E., respectively). In both cases, GI then decreased to a minimum in January (5.74 ± 0.59 S.E. for males, and 5.67 ± 0.40 S.E. for females, Fig. 2A). For females, mean oocyte size, measured as equivalent circular diameter (ECD; µm), increased from January to peak in July (74.4 µm ± 0.57 µm S.E.), followed by a decline and a subsequent increase again in September (71.8 µm ± 0.68 µm S.E.). Mean ECD then decreased from September to a minimum in January (46.3 µm ± 0.31 µm S.E., Fig. 2B). The proportion of gonad area dedicated to nutritive phagocytes (NPs) was inversely related to ECD (r^2^ = 0.45, p < 0.001), where, as oocytes matured, relative proportions of NPs decreased (Fig. 2C). However, as a proportion of GI, NPs increased in April to 60.8% ± 2.1% S.E. of GI, and then again in December to 79.6% ± 2.0% S.E. of GI. At its lowest, the proportion of GI accounted for by NPs declined to 49.5% ± 2.8% S.E. of GI in August (Fig. 3). The overall GI pattern with two peaks resulted from the combined variation in oocyte and NPs.

The proportion of males in spent and recovering maturity stages peaked in January (52.0% ± 13.2% S.E.), followed by increasing proportions of individuals transitioning to growing stages of maturity, peaking in May (75.7% ± 7.0% S.E., Fig. 2D). Following this peak, proportions of mature males increased from May to October/November (peaking at 87.7% ± 10.3% S.E. of individuals), from which point the proportion of spent and recovering individuals increased again until January (Fig. 2D).

### Changes in gonad index

The partials plot analysis revealed co-linearity between temperature and chlorophyll-a (Chl-a) (Pearson’s r = 0.73) and between salinity and temperature (Pearson’s r = 0.54). Therefore, based on a weighted importance analysis, only Chl-a was included in the model. The starting model fit (null model) was as per Eq. (1).

1 $$ {\text{Gonad}}\;{\text{Index}} = {\text{f}} \left( {{\text{Time}}} \right) + \upvarepsilon $$

where f is the smoothing function. This model had an AIC of 1368. Initially, single environmental variables considered as potentially ecologically influential, were added to the model. Following automated comparisons of all possible model variations, Chl-a was identified as the main environmental predictor of GI variation, with sex as a factor and as an interaction with the smoothed functions of time and Chl-a. Since other single environmental variables were not considered significant, the large-scale climate metrics, SOI and SAM, were included as additional factors to Chl-a and sex. There is some evidence that SOI and the SAM can influence each other^33^. However, the partials plot in our time-series did not reveal co-linearity between SAM and SOI. Other studies have also reported weak relationships between these metrics^34,35^. As such, we included both metrics in our model since both were significant factors in explaining some of the remaining variance in GI and together, improved the model AIC. It should however be noted that the SAM had the lowest relative importance comparative to other variables in the model (Supplementary information, Fig. S1). The final model resulted as per Eq. (2).

2 $$ \begin{aligned} {\text{Gonad}}\;{\text{Index}} & = {\text{f}} \left( {{\text{Time}}, {\text{by}} = {\text{Sex}}} \right) + {\text{f}} \left( {{\text{Chlorophyll}}, {\text{by}} {\text{Sex}}} \right) \\ & \quad + {\text{f}} ({\text{SOI}},{\text{by}}\;{\text{Sex}}) + {\text{f}}({\text{SAM}}) + {\text{Sex}} + \upvarepsilon \\ \end{aligned} $$

This model had an AIC of 1259, compared to the null model. This model was ranked highest with regards to AIC, and explained 41.4% of the variance in GI. Model predictions fitted well with the raw data and aside from the functions of time and sex, environmental covariates, Chl-a, SOI and SAM, were considered the best predictors of variance in GI, explaining 12.4% of the variance. The model met the assumptions of homogeneity of variance and model residuals were normally distributed. All Cooks distance values were < 0.034 and were not considered influential^36^.

**Figure 4:**
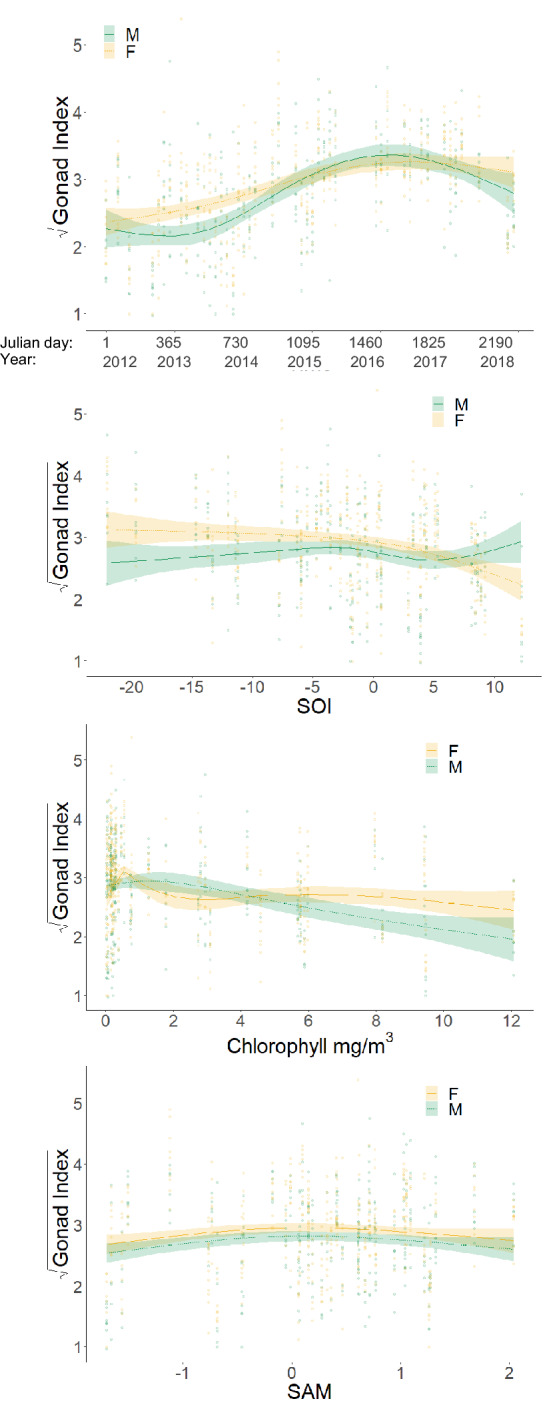
Smoothers of the effect of the three non-parametric terms: time, Southern Oscillation Index (SOI), chlorophyll and Southern Annular Mode (SAM), on the gonad index of *Sterechinus neumayeri,* from the optimal GAM model. Shaded area represents a 95% confidence interval and data points represent raw gonad index data. The magnitude of change in gonad index as a response to the change in the x-variable is represented on the y-axis as the square-root transformed gonad index. For the axis ‘time’, year intervals are plotted on every 1st January. Green represents males and yellow represents females.

The estimated covariate smoothers (Fig. 4) show there was a positive relationship for the smooth function of time, where both male and female GI increased from mid-2013 to mid-2016. Male GI then declined until the end of the time series suggesting a multiyear trend. The variation in GI explained by the model was limited by the individual GI variability within each month sampled. However, the model captures the overall increasing and decreasing trend observed over time.

For the covariate SOI, there were limited data for extreme negative SOI values of < −15, and so over-interpretation of the model predictions at this tail end was avoided. In comparison, data collected for SOI values > −5 were comprehensive and showed a negative association between GI and SOI for females when SOI was positive. This was not the case for males, where the relationship was not significant. For the covariate SAM, there was evidence of a bell-shaped curve in the relationship, with GI values peaking at SAM values of 0. Negative and positive SAM values resulted in lower GI. There was less certainty in the negative association between GI and positive SAM values due to the increase in the 95% confidence interval (CI) and the poor fit between the data points and the model prediction. As such, over-interpretation of this association was avoided.

**Figure 5:**
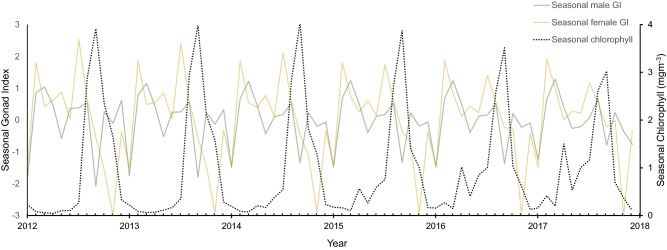
Seasonal cycle of gonad index for males (solid green line) and females (solid yellow line), extracted from decomposition analysis, overlaid on seasonal cycle of chlorophyll (dotted black line) extracted from decomposition analysis.

There was good data coverage at low Chl-a concentration because of the highly seasonal productivity. However, the association of GI with Chl-a concentration was negative for males from concentrations exceeding 1.5 mg m^−3^. This downward trend followed an initial increase in GI for both males and females at low Chl-a concentrations. This inverse relationship was also evident from the decomposition analysis, where seasonal cycles were extracted from both GI and Chl-a (Fig. 5).

Chlorophyll and temperature trends, extracted by decomposition analysis, showed positive linear relationships with SOI, where positive values of SOI (La Niña) correlated with high temperature (R^2^ = 0.207, p < 0.001) and Chl-a concentrations (R^2^ = 0.226, p < 0.001) (Supplementary information, Fig. S2 and S3). Other variables lacked significant linear relationships, lagged or unlagged, with the SOI trend.

### Oocyte growth and maturation

Oocyte mean diameters varied significantly between years for the months March-August and also for November and December, with March having the most interannual variability. For all other months, the mean ECD was not significantly different between years (Supplementary information, Table S3).

Size distributions of developing oocytes within individual females were bimodal for most months of the year (i.e., from February/March, through to October). In December oocyte sizes were mostly 6–80 µm. The oocyte distribution then broadened, with bimodal peaks appearing from January to February/March, and oocyte sizes ranging from 12–122 µm. In July, the frequency of large oocytes (80–135 µm), peaked at 51.1% ± 4.9% S.E., and steadily decreased until November, when they almost disappeared from the distribution accounting for 9.6% ± 2.5% S.E. of the distribution. Over this period, a cohort of small oocytes (12–80 µm) increased in frequency from 49.0% ± 4.9% S.E. in July, through to 90.4% ± 2.5% S.E. in December (Supplementary information, Fig. S4).

### Male maturity

**Figure 6:**
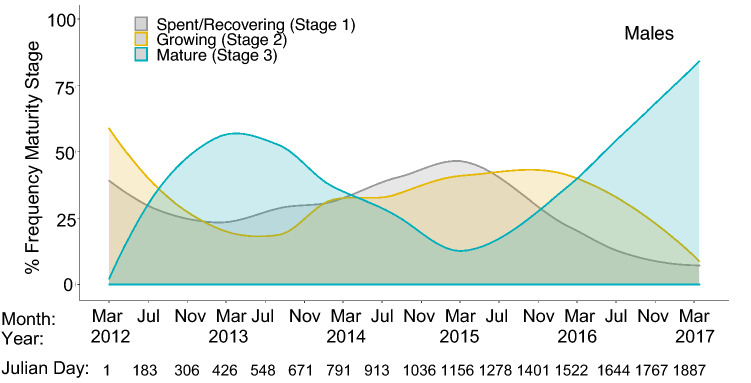
Long-term changes in the percentage frequency (%), represented as a density plot, of maturity stages in the male sample population from March 2012–March 2017. Frequency densities derived from (LOESS) method. The smoothing span was chosen to reflect long-term changes rather than seasonal variability.

Males varied in reproductive maturity on both seasonal and interannual scales. Substantial individual variation was evident since the maturity categorisation was broad, with 105 ± 20 S.D. individuals in each category emphasising the large variation. Across the time-series, from March 2012–2017, there were periodicities in the proportion of maturity stages present (Fig. 6). A distinct bell curve in the proportion of mature males occurred from March 2012–2015, peaking in March 2013. This distribution overlapped almost simultaneous bell curves in spent/recovering and growing stages, which peaked in March 2015 and November 2015, respectively.

## Discussion

Data presented here comprise the longest reproductive time series of an Antarctic benthic marine invertebrate to date and are the first to provide evidence that reproductive allocation may be accumulated across a multiyear scale with no evidence for an environmental link. Where multiyear studies have been conducted, evidence is building that some marine invertebrates have endogenously driven growth and reproductive cycles, in which environmental variation is independent, or interacts with this internal rhythm to regulate reproductive investment and growth over time^1,22^. Our model outcomes suggest a multiyear trend in reproductive investment which could suggest an innate endogenous rhythm. Owed to the number of years comprising the time series, repetition of this trend has not been captured in our dataset and therefore it is necessary to interpret these results with caution. However, several studies have recorded multiyear cycles of similar periodicities in a range of Antarctic species. For the crab-eater seal around the Antarctic Peninsula, the juvenile leopard seal at Macquarie Island, and the Weddell seal in McMurdo Sound, 4–5 year intervals for both reproduction and population peaks have been identified^21^. Other Antarctic research has shown that some bivalves exhibit endogenous growth cycles, with a 9.06 year cycle present for *Aequiyoldia eightsi*, and two endogenous cycles of 5 and 6.6-years reported for *Laternula elliptica*^22^. There is therefore evidence for a temporal mediator of growth and reproductive investment in Antarctica, possibly because of low temperature effects on biological rates and extreme seasonality, where it may take multiple years to build reserves, with seasonal spawning and growth taking place against a backdrop of long-term investment over several years.

The very small number of multiyear investigations suggests endogenous cycles in growth and reproduction may be more common than previously identified. A similar trend to that identified in GI was also evident in our male maturity data, where we observed a single peak in each maturity stage across a 5 year period, with the ‘growing’ phase of maturity, matching the shape of the curve for variation in male GI. Extreme interannual variation in reproductive condition and a circaseptennial rhythm (7-year cycle) has been previously proposed for *S. neumayeri*^28^ based on studies of the Pacific purple sea urchin, *Strongylocentrotus purpuratus*^37^*.*

Alongside the temporal signal in GI, our data also indicate that there is environmental entrainment of this periodicity as the whole population builds gonad and spawns synchronously across the cycle. This entrainment likely arises from a combination of factors. However, our analysis identified a negative relationship in female GI when the SOI signal is positive (La Niña) and negative relationship in both male and female GI when the SAM signal is negative. Climate coupled atmospheric-oceanic-sea-ice processes, like SOI and SAM, are known to impact ecosystem variability in Antarctica, especially for the Antarctic Peninsula^38–40^. These effects could result from direct links caused by changes in physical factors including ocean temperature, or else indirect links resulting from altered primary productivity and food web dynamics^35,41,42^. Effects could also result from a combination of, or synergistic interactions between, factors. Isolating a single cause and effect of these large-scale climate indices is challenging, when many environmental factors are closely linked and the consequences of change in one variable cascades through multiple physical and biological pathways^43^.

Relationships between SOI and climate parameters in Antarctica are non-linear, but there is strong evidence that El Niño and La Niña signals are translated widely across the southern hemisphere^12,44^. Here La Niña episodes have been correlated with warm SST anomalies and decreased sea-ice extent, while El Niño episodes produce opposite effects^20,45^. Our time series encapsulates El Niño years up to values of -20, and La Niña values of +10. Our model predictions indicate a negative association with reproductive investment for females when SOI is positive (La Niña), and a positive association when SOI is negative (El Niño). Reasons for this are unclear, however our regression analysis of Chl-a and temperature against SOI, suggest that positive SOI correlated with higher temperatures and higher chlorophyll concentrations, outside the usual seasonal variation. These relationships would likely have implications for sea-ice cover and water column stratification^17,46^. Because of this interaction of factors, the SOI can be used to highlight extremes in the environment and multifactorial shifts, rather than that of a single mechanism driving an ecological response.

The drop in GI at positive SOI (La Niña) is only present in females. Reasons for this could be owed to a higher energy requirement for oocyte development^47,48^. Therefore, reproductive costs for females may be higher and trade-offs in energy allocation to reproduction may be necessary during periods of environmental change^49^. Possible changes in environmental variables as a result of La Niña events may then result in a reduction of GI indices for females only.

Impacts of large-scale climate metrics on Antarctic biodiversity have been identified across a range of taxa. Mammals including Weddell seals and Elephant seals have reproductive rates in phase with the SOI^21,42^. Other studies have shown strong links with plankton population abundance and krill reproductive recruitment success^38^, and seasonal vertical migration behaviour^13^. Abundance of the planktonic tunicate, *Salpa thompsoni* has also been correlated with SOI^46^*,* as well as 5-year cycles in abundance peaks for krill, *Euphausia superba,* where high abundance was associated with greater sea-ice extent^50^. The authors are unaware of any studies that demonstrate such correlations for Antarctic benthic species or SOI effects on species or ecosystems this far south.

Interestingly, studies have shown that the SAM is more closely linked to interannual sea surface temperature variability around the WAP, compared to SOI^35^. Our model exploration shows that the SOI accounts for more of the variability in the GI of *S. neumayeri* than the SAM, and both temperature and SAM had low relative importance in predicting GI. These results further suggest that temperature is likely not the most important or only driver of reproductive processes in these thermally sensitive species, and instead there is more complexity underlying the interaction between large-scale climate metrics, local environmental drivers, and biological functioning.

Temperature and Chl-a variation can be seasonal drivers of reproductive cycles^7,8^. Our results show that Chl-a concentration co-varies with reproductive condition in *S.neumayeri*, where the negative relationship between GI and Chl-a alludes to spawning being correlated with Southern Ocean summer phytoplankton characteristics^51,52^. Model predictions show that when Chl-a concentrations increase, GI declined, which is indicative of spawning. The relationship between GI and Chl-a may be indirect, whereby spawning is initiated prior to the bloom either by a trigger associated with early phytoplankton increase to facilitate feeding of planktonic larval stages^31^ or another environmental factor co-varying with Chl-a. This hypothesis would also result in a negative association between trends in GI and Chl-a. Again, we see different responses for males and females to high Chl-a concentrations. Since the GI relationship with Chl-a is likely a result of spawning, it may be that we observe a smaller reduction in GI in females following spawning due to the presence of nutritive phagocytes (NPs). We provide evidence that NPs are still present in the gonad following spawning, and hence will contribute to the higher GI post-spawning. Although we do not have NP data for males in this study, for the temperate sea urchin, *Strongylocentrotus droebachiensis*, the volume of NP in males was lower than in females throughout the gametogenic cycle^53^. If NPs contribute less to the GI in males, it is reasonable that we would see a larger decline in GI following spawning.

Seasonal patterns in *S. neumayeri* reproduction were evident in GI, male maturity and oocyte size distributions, all of which exhibited periods of maturation and growth, followed by phases that implied spawning across several months (e.g., decreasing GI, decreasing mean oocyte size for females, or increasing spent/recovering stages for males). Monthly changes in GI during the year highlight this reproductive seasonality. However, for urchins, gonad tissue comprises both germ cells (gametes) and NPs^54,55^. Our data demonstrate that fluctuations in female GI are caused by both the maturation of gametes and changes in gonad proportions dedicated to NPs. These changes are clearest in April, where GI increases significantly from March. However, this GI increase is due primarily to larger NP increases, rather than oocytes. The April GI peak for females follows the end of the summer phytoplankton bloom and suggests the resultant phytodetrital pulse to the seafloor is the primary nutritional source for this species. This explanation is further supported by fluctuations in *S. neumayeri* gut mass during the season. Gut mass decreases during the first half of the austral winter, when feeding activity ceases, followed by stabilisation until the onset of feeding in November^30^. Gut index data for *S. neumayeri* in 2017/18 also supports this finding (Supplementary information, Fig. S5). NPs accumulate in late summer and early winter. During the period of cessation of feeding in winter, NPs transfer nutrients to developing gametes. NP stores are also used to meet the urchin’s energy requirements for metabolic maintenance during this time^56^. Our results suggest these nutrient stores are important reproductive reserves in *S. neumayeri* as proportions vary inversely with mean oocyte size and NPs are depleted as reserves are mobilised to maturing gametes.

This research demonstrates the need for long-term multiyear studies that encapsulate endogenous and environmentally driven reproductive investment against a backdrop of seasonal change. Relationships between reproductive cycles and single environmental variables are well reported, where spawning often coincides with seasonal changes (e.g., in temperature and chlorophyll). However, gradual environmental shifts over several years are rarely encompassed by single variable studies. Furthermore, it is more likely that such change occurs from alterations in multiple interacting variables. Large-scale climate metrics (e.g., SOI and SAM), can capture shifts in multifactorial environmental states and highlight how environmental alterations translate into ecological processes.

Identifying endogenous rhythms for growth and reproduction enables the partitioning of these processes from the effects of small-scale environmental change and large-scale environmental cycles on marine biodiversity. We have identified a potential multiyear trend in reproduction in a polar sea-urchin, *Sterechinus neumayeri,* from a seven year dataset. There may be even longer cycles, and cycles like these may be cumulative across decades or multi-decadal timescales. To identify such long-term trends requires very long sampling and monitoring programmes, but these would be invaluable when assessing the impacts of the current environmental change that is occurring over decadal to centennial scales.

## Methods

### Study site and sampling

*Sterechinus neumayeri* were collected from Hangar Cove (67°33′54.2′′S 68°07′13.1′′W), located near the British Antarctic Survey’s Rothera Research Station on the Western Antarctic Peninsula (Fig. 1). Adult urchins (19–51 mm diameter; n = 16) were collected monthly (weather permitting) from 2012 to 2018 by SCUBA divers (13–21 m depth), with the exception of a 6-month gap from August 2015 to January 2016, when thin ice prohibited access to the collection site. Specimens were preserved in 10% buffered formalin solution until analysis.

### Measuring reproductive condition

GI, oocyte size and tissue composition in females and maturity stage in males were used to describe urchin reproductive condition. Total gonad wet mass was measured and water content and dry gonad mass obtained from subsamples of gonad tissue. GI was used instead of direct gonad mass to allow for differences in animal size, and was derived by calculating the gonad mass as a proportion of total body size according to Eq. (3), following Bronstein et al. (2016):

3 $$ {\text{GI}} = \frac{{{\text{Total}}\;{\text{gonad}}\;{\text{dry}}\;{\text{mass}}\;({\text{mg}})}}{{{\text{Test}}\;{\text{diameter}}\;({\text{mm}}) }} $$

A subsample of wet gonad tissue was examined for oocyte size and tissue composition for females, or maturity stage for males following standard wax histology procedures^58^. In brief, tissue was dehydrated in a graded isopropanol series, cleared in XTF clearing agent, embedded in paraffin wax, sectioned at 7 µm and stained with haematoxylin and eosin.

Individuals were sexed and female tissue sections viewed under a light microscope (Olympus BHS (BH-2)) at × 10 magnification and photographed using a Nikon D5000 camera (Supplementary information, Fig. S6). To obtain oocyte size data, outlines were drawn around representative oocytes in images using imaging software, Fiji (image-J v2)^59,60^. Only oocytes with a visible nucleus or nucleolus were measured to ensure oocytes were centrally sectioned and maximum circumferences measured. Where possible, at least 5 females were analysed each month and 100 oocytes measured at random per female. Subsamples of 100 oocytes were used to calculate an average oocyte size distribution^28^. Oocyte area (A) was used to calculate the ECD according to Eq. (4), used in previous studies^61,62^ to determine the size of a spherical oocyte with an equivalent area.

4 $$ {\text{ECD}} = \sqrt {\frac{{4{\text{A}}}}{\uppi }} $$

Male tissue sections observed under light microscope at × 10 magnification were staged for maturity based on the development of the testes. Testis maturity level (Supplementary information, Fig. S7) was categorised from representative images following^2^:

Stage 1: Spent/Recovering: Lumen empty. Nutritive phagocytic tissue lining is of variable thickness and possibly a thin layer of spermatogonia on the germinal epithelium.

Stage 2: Growing: Spermatogonia visible on germinal epithelium; spermatozoa present at moderate density in lumen.

Stage 3: Mature: Lumen densely packed with mature spermatozoa in swirls. Lumen stains intense blue. Spermatid production may still be evident. Nutritive tissue generally highly reduced.

To visualise changes in male maturity as a continuous variable, both seasonally and across years, the occurrence of each stage was converted to a percentage frequency for each month ((number of individuals at given stage/number males sampled in month) × 100). Percentage frequency of maturity stage was then modelled as a smoothed function for month, using the local regression smoother (LOESS) method^63^.

### Nutritive phagocytes (NPs)

Sea urchin gonad tissue serves two functions. Tissues contain both the developing gametes and NPs, a storage tissue. Variation in GI is thus a product of variation in both tissue types and not limited to maturing gametes. Understanding how the proportions of these tissues change seasonally is important to understanding gonad function and interpreting seasonal GI variation. Images taken for oocyte size were, therefore, used to quantify proportions of NPs to oocytes in female gonads. For this purpose, three females from each month were selected at random, where for each, three images of histological sections from different areas in the gonad were used to provide a representative assessment. The relative areas occupied by germ cells and NPs were calculated using Fiji (image-J v2) ‘Area’ tool. This process involved first selecting only NPs as defined by specific colour thresholds and converting these areas to a mask. The mask % area relative to the image was then calculated. This process was also applied to oocytes. Gonad tissue was almost exclusively formed from oocytes and NPs, hence % area of NPs and oocytes was calculated relative to the total gonad tissue area in each image.

To relate NP and oocyte % areas to gonad size, and inform how these proportions contribute to seasonal GI variation, NP and oocyte proportions in the gonad were averaged across individuals for each month using images that were of highest quality from across the time-series. Following this, NP and oocyte percentages were scaled to represent relative proportions of the corresponding GI (averaged for all females for each month). Gonad oocyte and NP proportions were calculated based on % area tissue coverage estimates from image analysis according to Eq. (5).

5 $$ \begin{aligned} \left( {\frac{{{\text{Ao}}}}{{{\text{Ao}} + {\text{Anp}}}}} \right) & = {\text{AAo}}; \left( {\frac{{{\text{Anp}}}}{{{\text{Ao}} + {\text{Anp}}}}} \right) = {\text{AAnp}} \\ \overline{{\text{AAo }}} \times \overline{{{\text{GI}}}} & = {\text{Oocyte}}\;{\text{proportion}}\;{\text{of}}\;{\text{the}}\;{\text{gonad}} \\ \overline{{\text{AAnp }}} \times \overline{{{\text{GI}}}} & = {\text{Nutritive}}\;{\text{phagocyte}}\;{\text{proportion}}\;{\text{of}}\;{\text{the}}\;{\text{gonad}} \\ \end{aligned} $$

where Ao = Percentage area of oocytes; Anp = Percentage area of nutritive phagocytes; AAo = adjusted area of oocytes relative to gonad tissue total area; AAnp = adjusted area of nutritive phagocytes relative to gonad tissue total area; $\overline{{\text{AAo }}}$ = monthly mean of AAo; $\overline{{\text{AAnp }}}$ = monthly mean of AAnp; $\overline{{{\text{GI}}}}$ = monthly mean of female GI.

### Environmental covariates

Environmental data were collected weekly from Ryder Bay (67°34′12.0"S 68°13′30.0"W), ~ 4 km west of Hangar Cove. This oceanographic sampling regime is an on-going part of the Rothera Oceanographic and Biological Time Series (RaTS) that has run continuously since 1997^64,65^. Data from March 2012 to March 2018 were obtained for physiological drivers including temperature and salinity (at 15 m depth), as well as sea-ice extent and Chl-a concentration (a proxy for food availability). The regional climate metrics, SOI and SAM, were also considered as a covariate measure. SOI was represented as the standardised anomaly of the mean atmospheric sea level pressure (MSLP) difference between Tahiti and Darwin (Australian Bureau of Meteorology), calculated according to Eq. (6).

6 $$ {\text{SOI }} = {10 }\frac{{{\text{Pdiff}} - {\text{Pdiffav}}}}{{\text{SD(Pdiff)}}} $$

where *Pdiff* = (average Tahiti MSLP for the month)—(average Darwin MSLP for the month); *Pdiffav* = long-term average of *Pdiff* for each month, *SD (Pdiff)* = long-term standard deviation of *Pdiff* for the month in question.

SAM was represented as the standardized 3-month running mean value of the Antarctic Oscillation index, reported by NOAA National Weather Service Climate Prediction Centre.

### Data analysis

Data were initially tested for normality and homogeneity of variance, and identification of outliers and between-variable relationships, as per Zuur et al. (2007). A chi-squared test was used to assess whether sex ratios deviated from 1:1. A t-test was used to determine differences in size (i.e. test diameter and whole animal mass) and GI between males and females. An analysis of variance (ANOVA) was also used to determine differences in oocyte size between months of comparable gametogenic maturation/ stage across the time series (i.e., between years). Where significant difference were found (p < 0.05), the ANOVA was followed by a post-hoc Tukey pair-wise test.

An initial linear regression model of GI with time revealed patterns in the residuals, indicating an underlying non-linear relationship. Following this, a generalised additive model (GAM) was used to examine factors influencing reproductive state^66^. The response variable, GI, was used as a measure of reproductive state and modelled against smoothed ecological and environmental variables. Time, Chl-a, temperature, fast ice concentration, salinity, SAM and SOI were considered in the model as continuous predictors. Month and season were considered as factors accounting for seasonal periodicities and modelled as main effect predictors using cyclic cubic regression splines, and finally sex was considered both as an interactive and main effect predictor. In order to meet the assumptions of normality, GI was square root transformed.

An initial pair’s plot was constructed to determine co-linearity between predictor variables before adding them to the model. Of the explanatory variables that correlated (threshold correlation for inclusion = 0.50), the most ecologically relevant variable was included in the initial model. If both variables were ecologically relevant, then the weakest predictor was removed. Penalised cubic regression splines were used to estimate the smooth function for each non-cyclic predictor variable and with knots limited to 5 which was deemed adequate to explain the data, without over-fitting^67^. Using the “FSSgam” package in R^68^ a full-subsets information theoretical approach was used to compare a complete model set of all predictor variables from the environmental and ecological data available. Other relevant R packages for the model included the “MuMin” and “mgcv” packages^69^.

All candidate predictors were considered during the initial model exploration and ranked in order of conditional probability, calculated by the Akaike Information Criterion (AIC). Variable weights were ranked by importance and predictors ranking low were excluded. Residuals from the ‘best’ model were checked for normality and homogeneity of variance using the “gam.check” function in the “mgcv” package.

Decomposition analysis was carried out on the GI time series data as an alternative method, to identify any overall trends and seasonal components. This analysis produced results very similar to the GAM model and did not provide any additional information. However, this additional analysis did substantiate the GAM model results (Supplementary information, Fig. S8). Decomposition of the environmental variables was also explored and trends, seasonal cycles and residual variations were observed in relation to the GI trend. Decomposition of the environmental variables allowed the trends observed for each factor to be regressed against SOI to determine how this large-scale climate metric might relate to single variables measured in our time series. Lag effects were also considered and incorporated where visualisation of the trends alluded to a delay in biological response. The R code for this analysis is given in the Supplementary information (Supplementary information, Text S1).

## Supplementary Information

Supplementary Information.

## Data Availability

Data available from the Dryad Digital Repository https://doi.org/10.5061/dryad.6q573n5z1^70^.
