# Supplementary material for: Multiyear trend in reproduction underpins interannual variation in gametogenic development of an Antarctic urchin
Source: Sci Rep. 2021 Sep 22;11:18868. doi: 10.1038/s41598-021-98444-4 (PMC8458454; doi:10.1038/s41598-021-98444-4)
Supplement: Supplementary file 1 — Supplementary Information. [file 41598_2021_98444_MOESM1_ESM.docx]

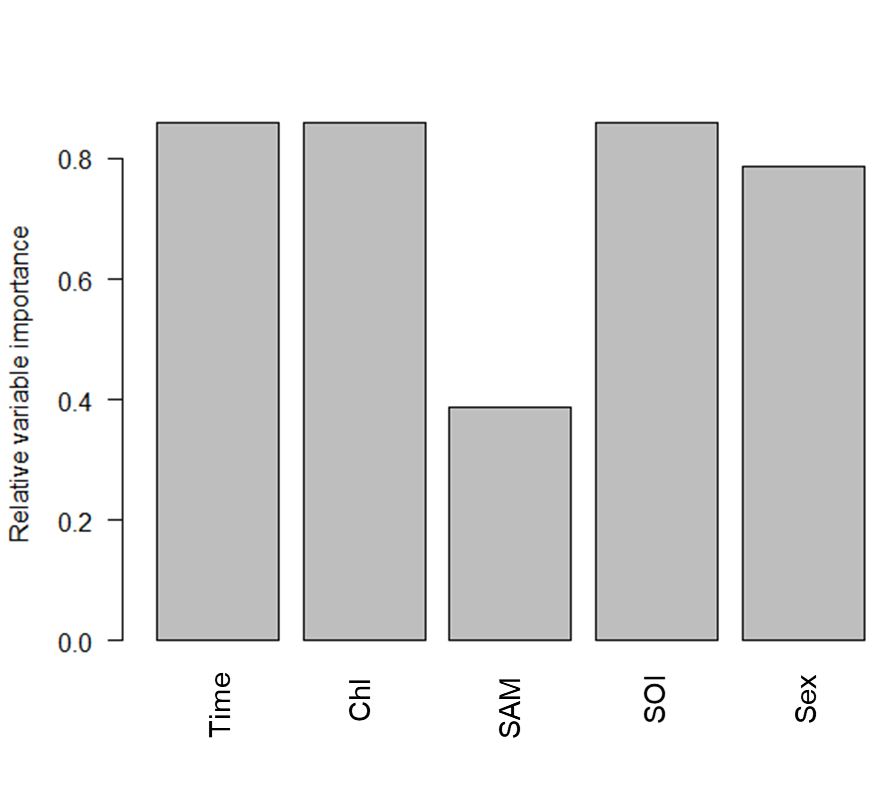
Figure S1: Relative variable importance of large-scale climate metrics and single variables in final general additive model exploration. Chl = Chlorophyll, SAM = Southern Annular Mode and SOI = Southern Oscillation Index.


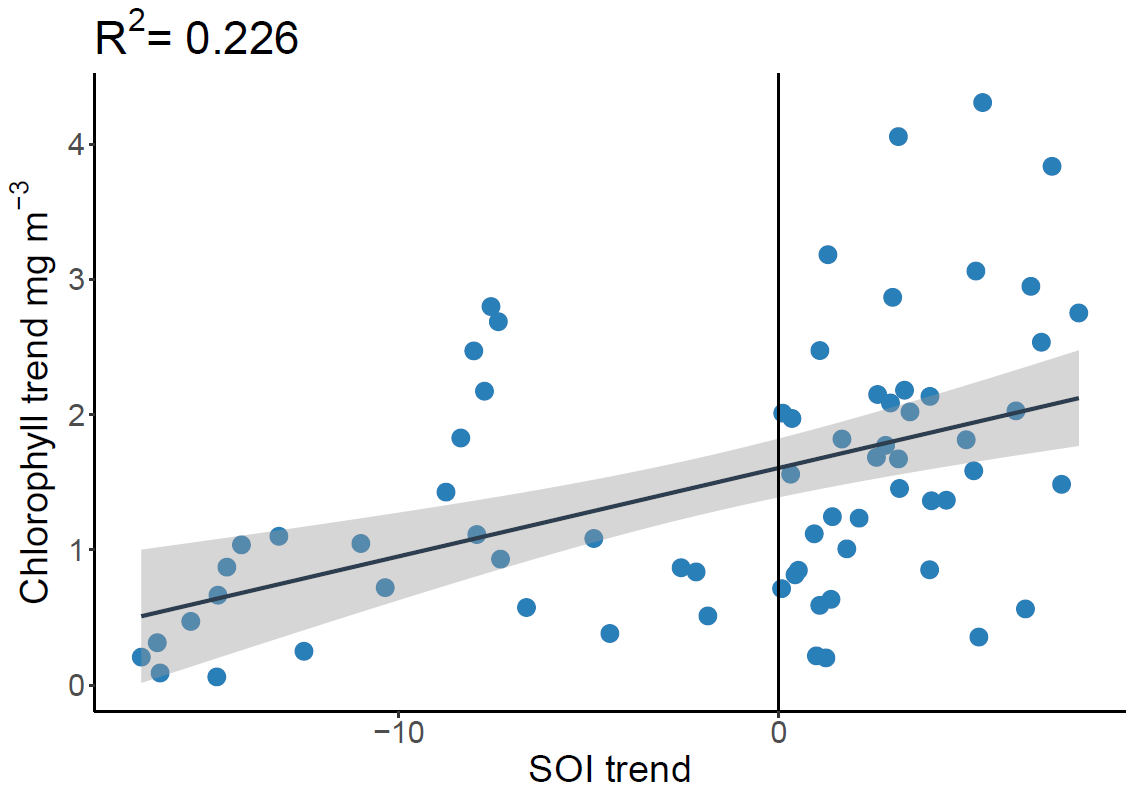
Figure S2: Linear relationship between the SOI trend data and the temperature trend data from 2012 – 2018, extracted from the decomposition analysis.


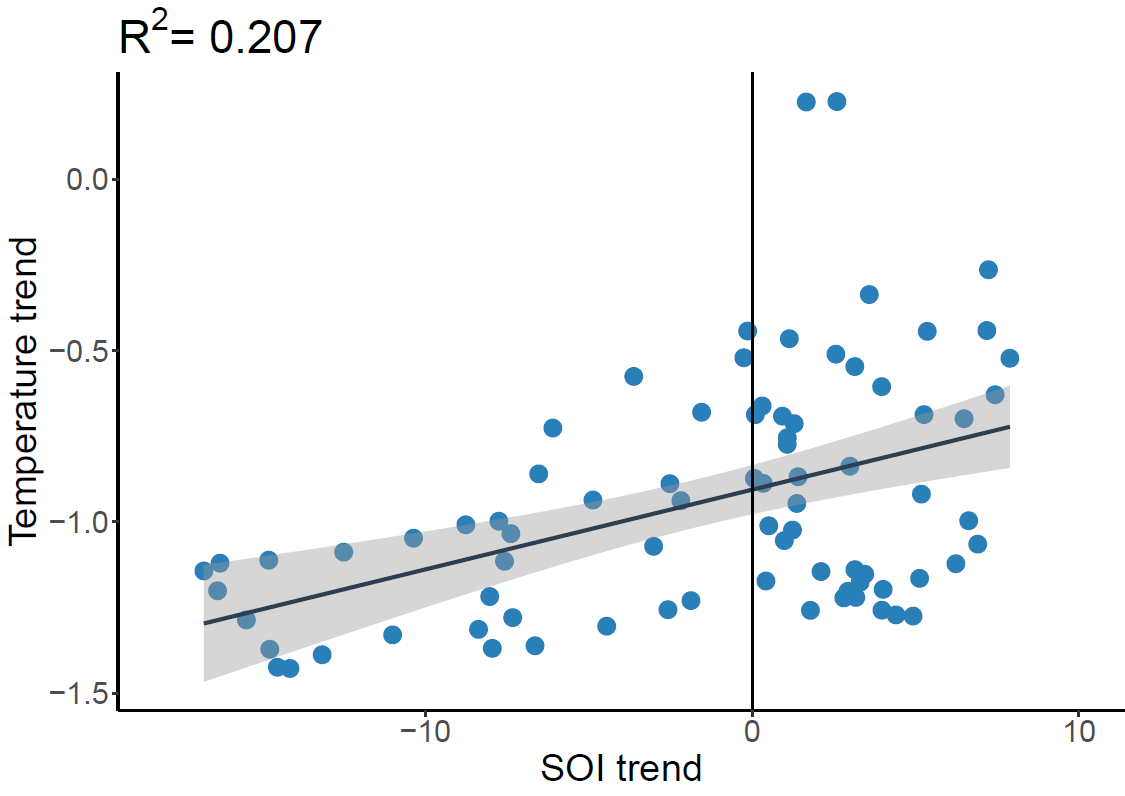


(^o^C)

Figure S3: Linear relationship between the SOI trend data and the chlorophyll trend data from 2012 – 2018, extracted from the decomposition analysis.

Figure S4: Oocyte size distributions (based on the calculation of Equivalent Circular Diameter, µm) displayed as monthly histograms of female oocyte size percentage frequencies (%). From left to right, years are displayed from the time series from 2012 to 2018. From top to bottom, months are displayed from January to December. Error bars are ± standard error. N = total number of females included in the distribution, n = total number of oocytes measured in the distribution.


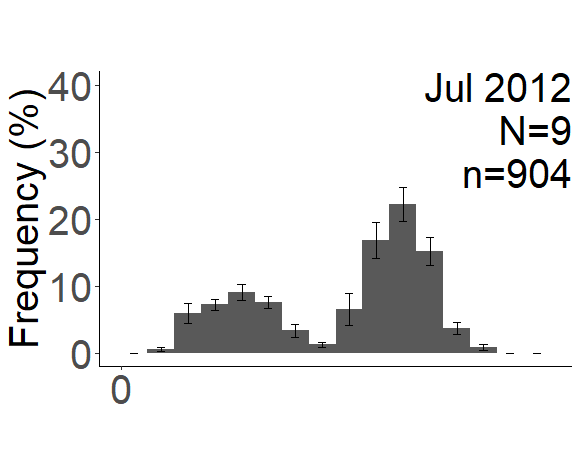

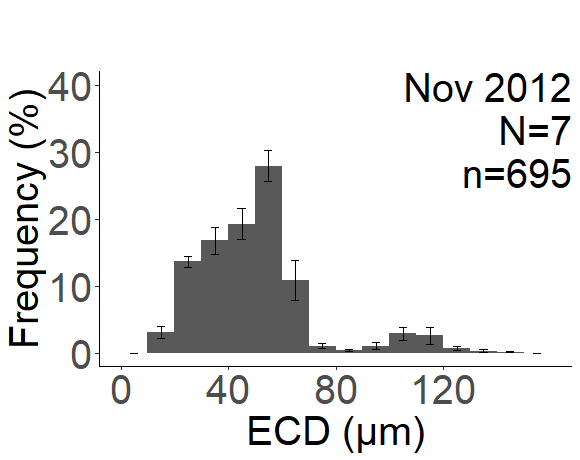

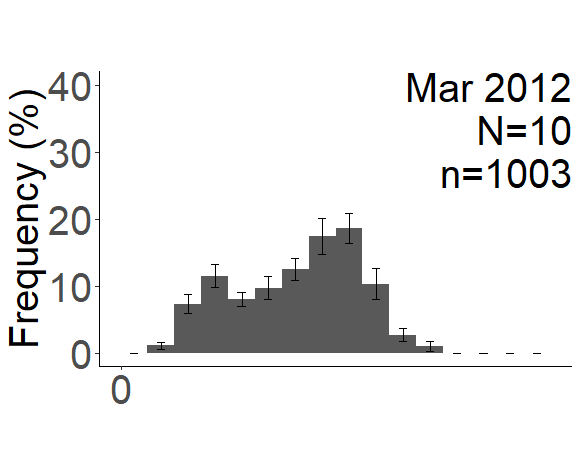

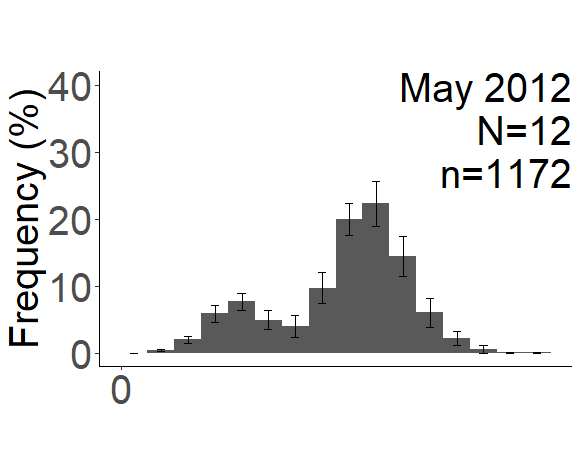

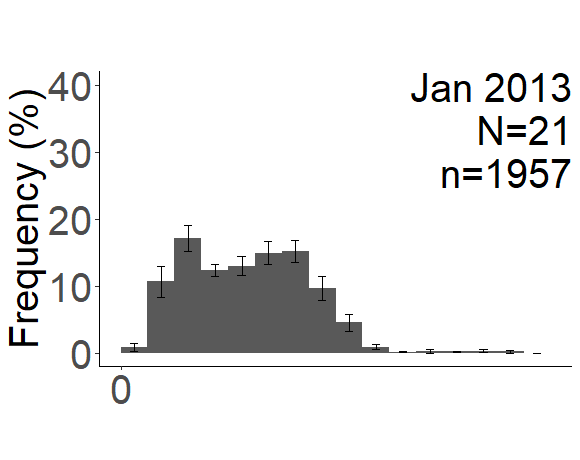

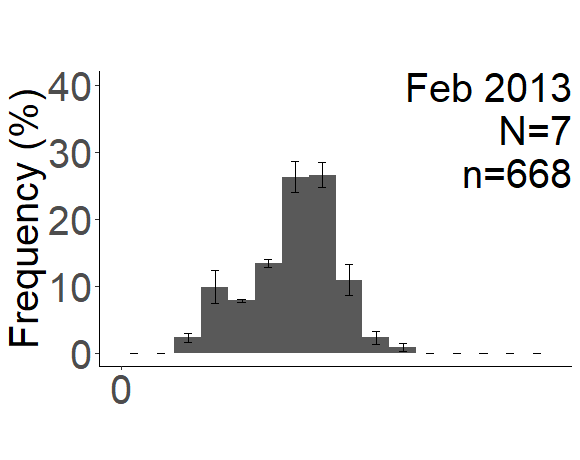

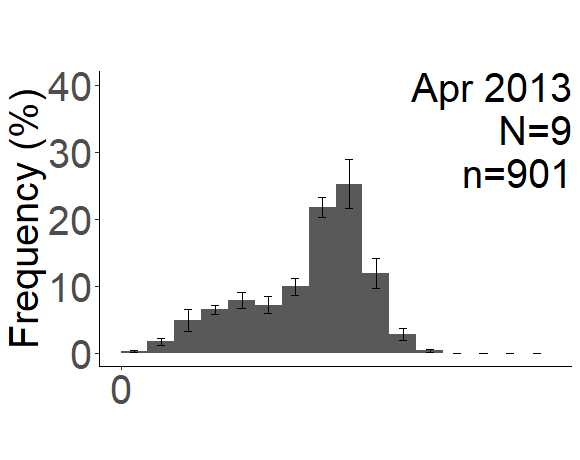

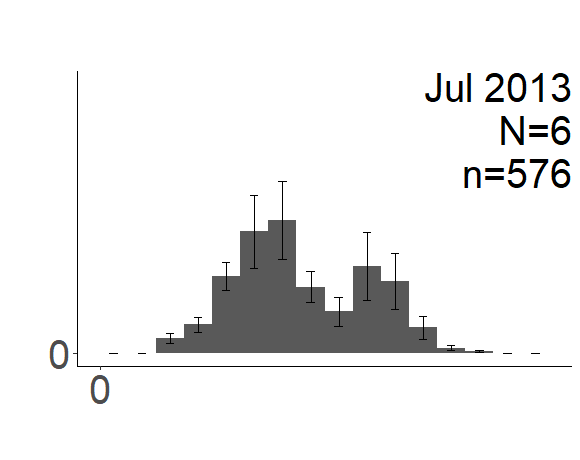

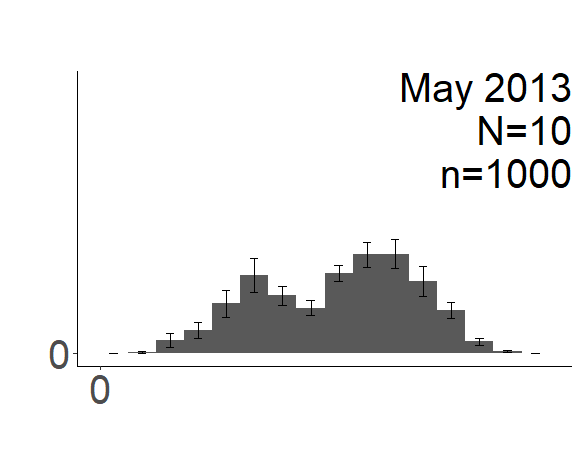

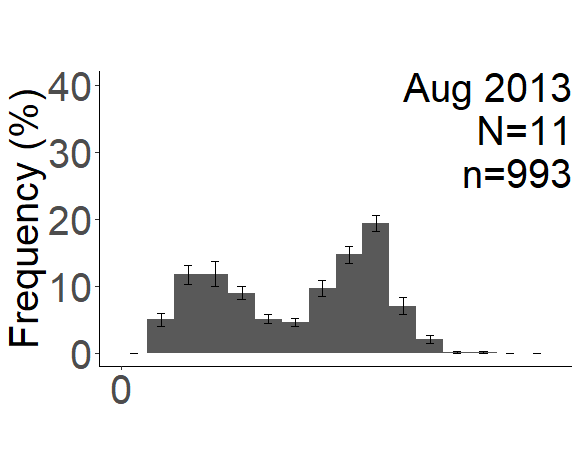

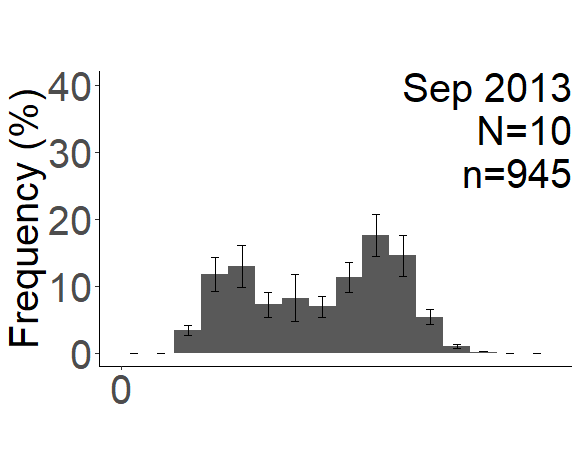

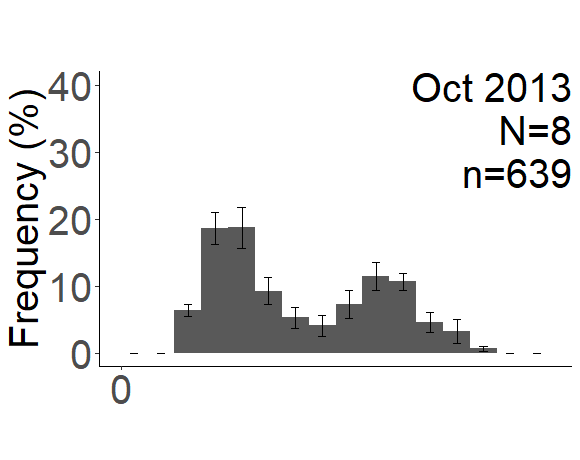

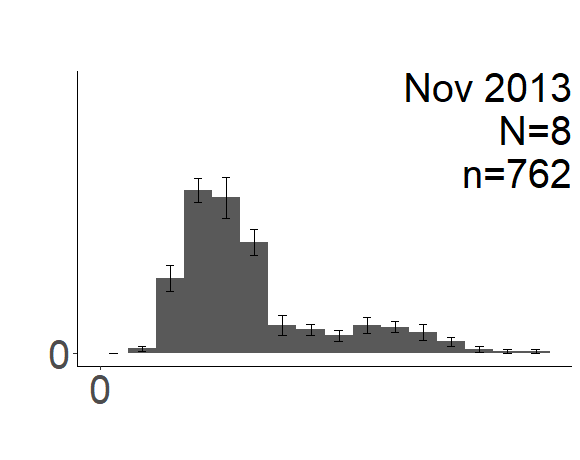

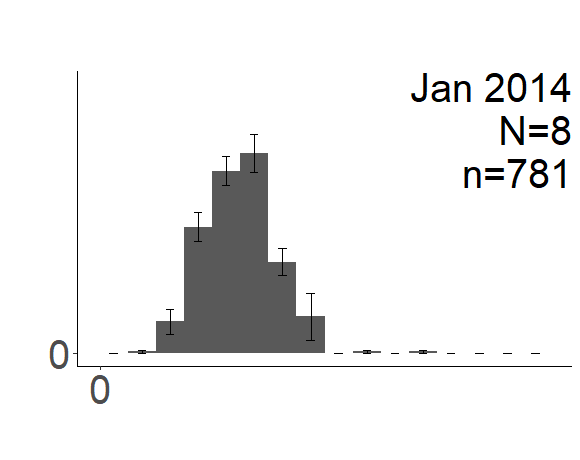

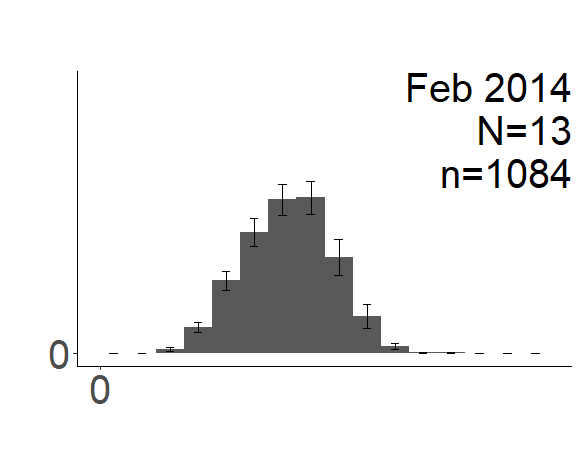

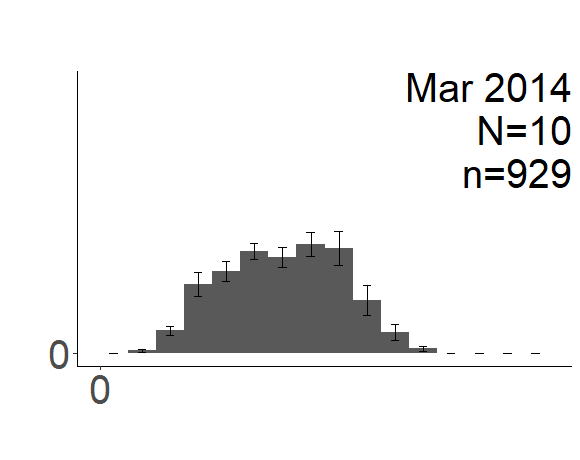

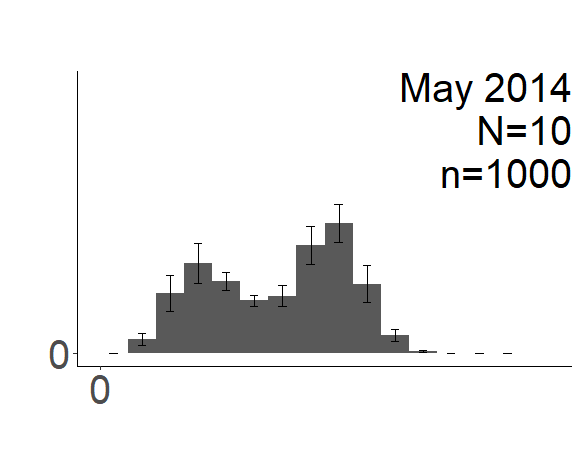

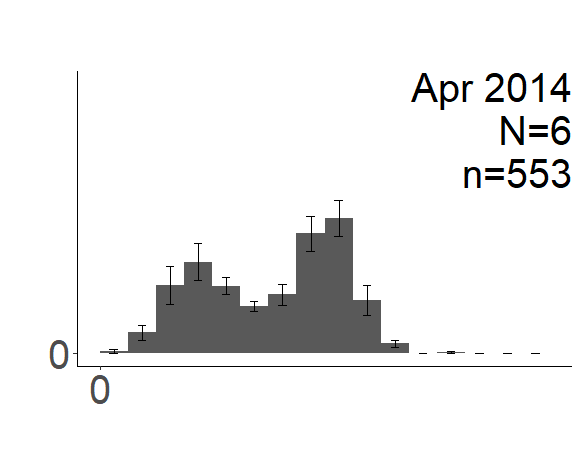

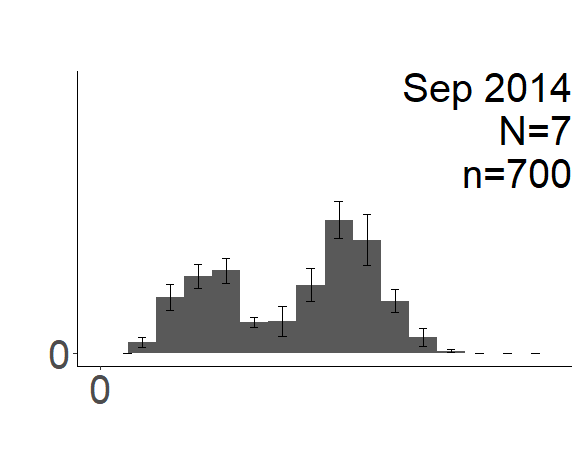

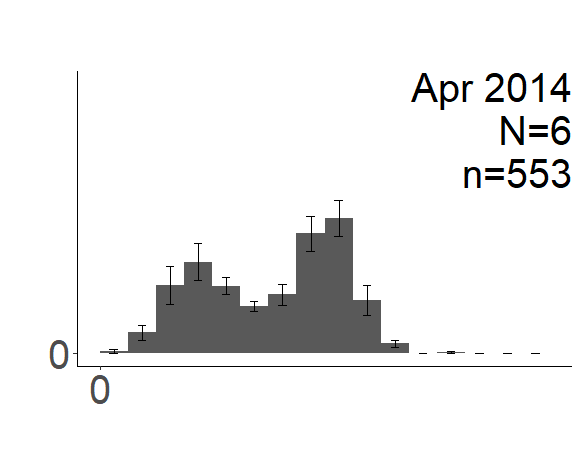

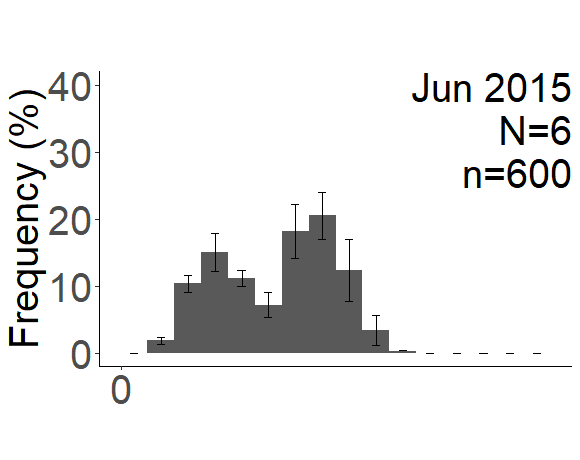

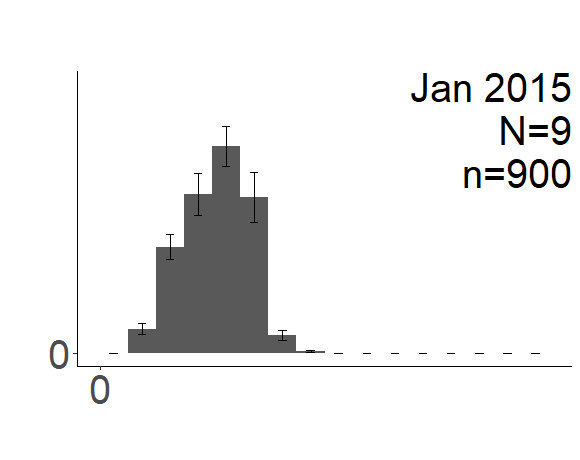

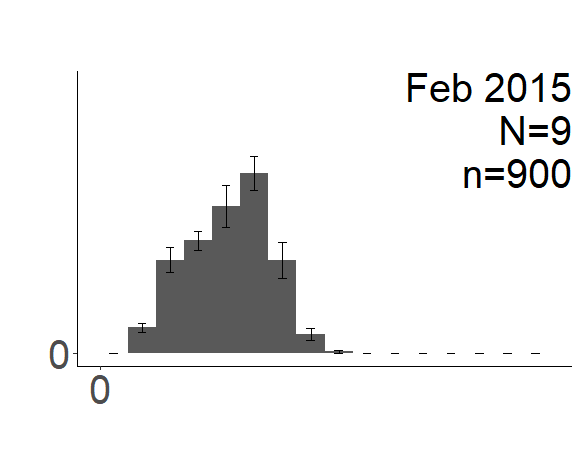

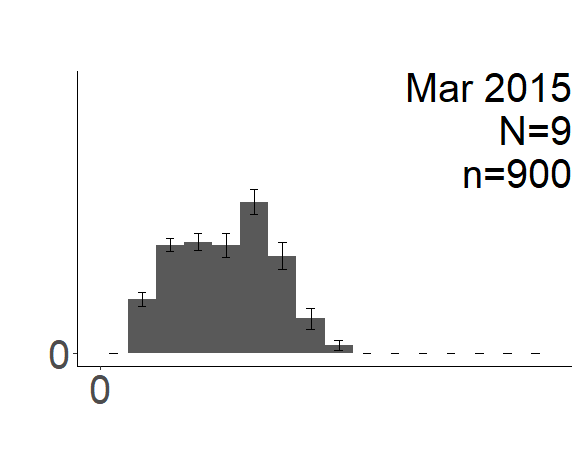

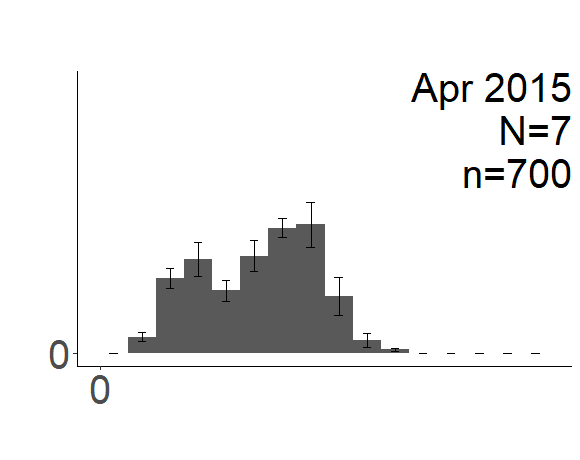

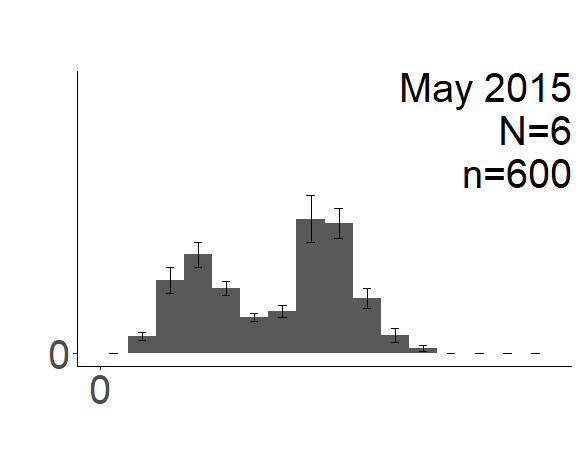

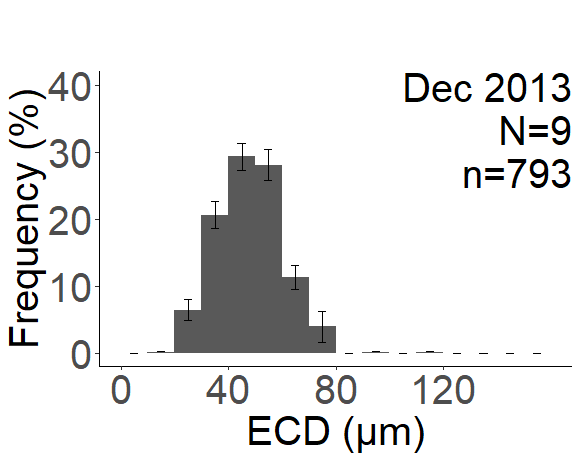

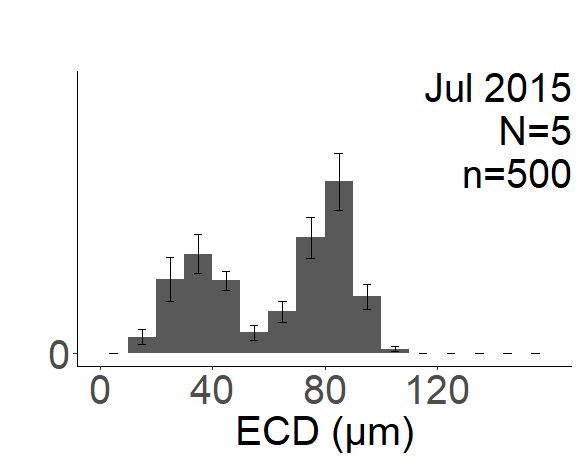

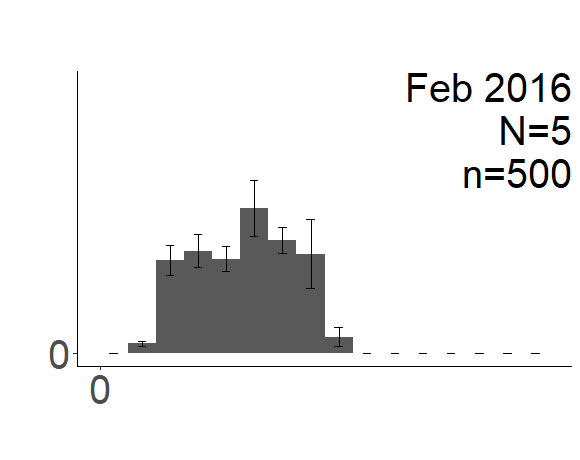

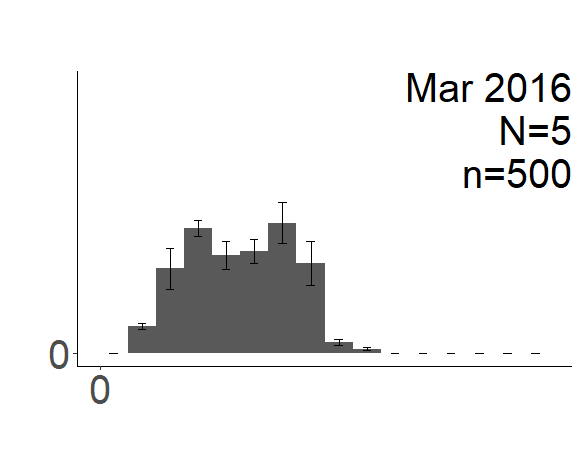

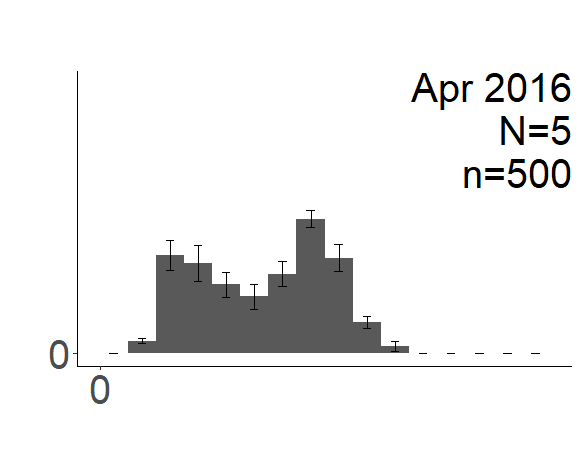

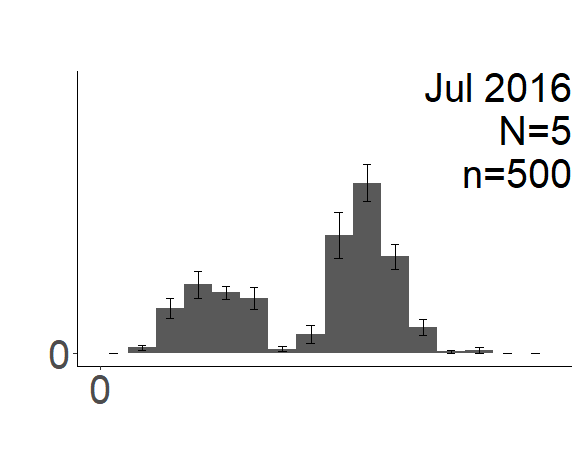

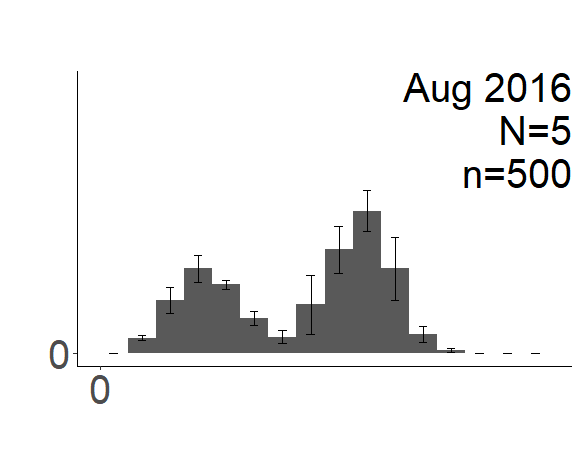

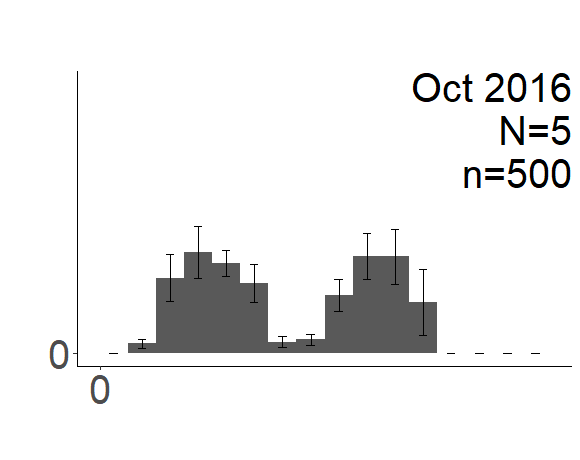

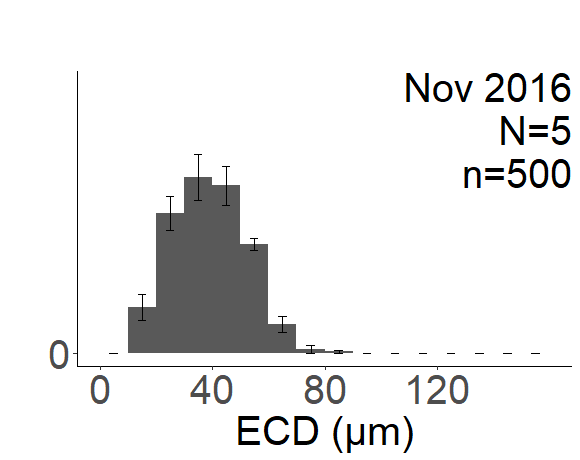

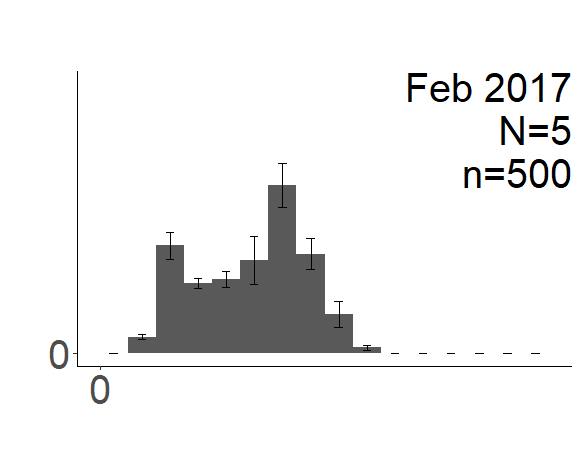

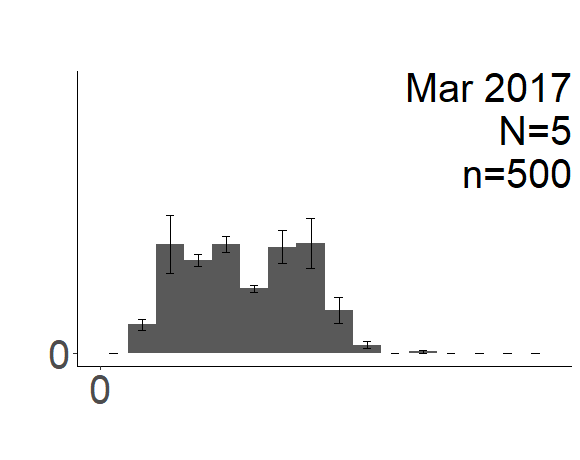

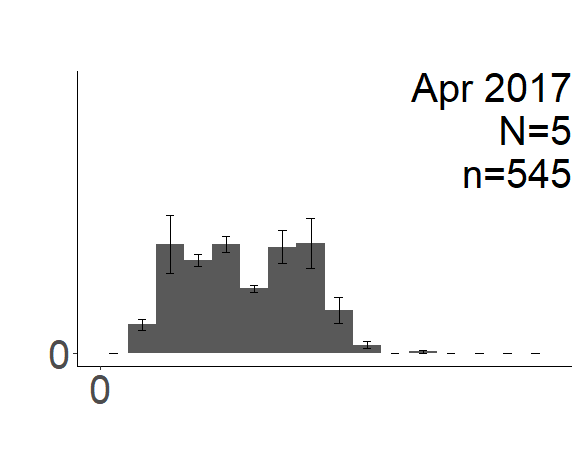

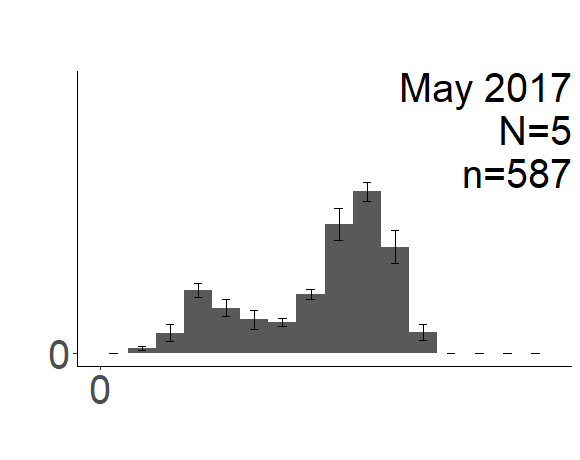

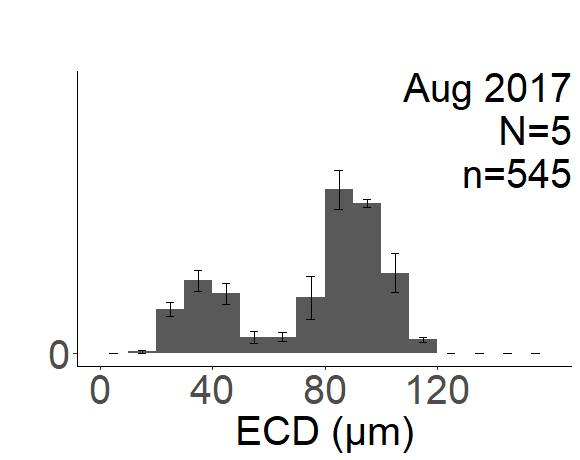

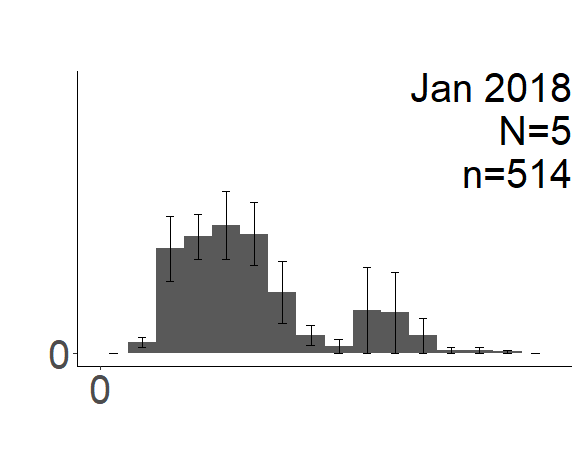

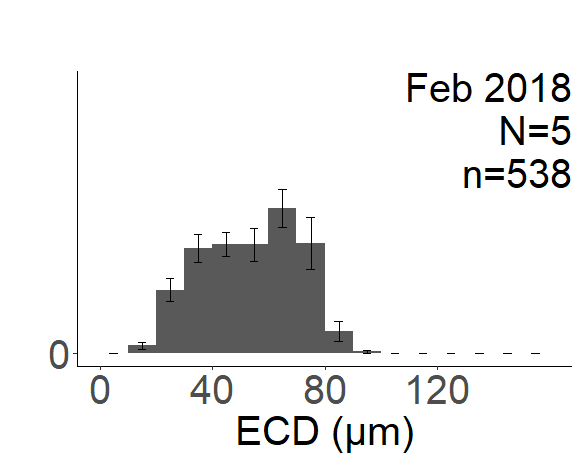


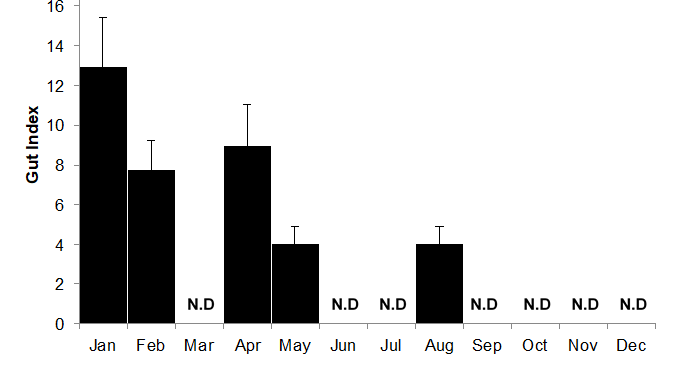
Figure S5: Gut index ((*gut mass/total animal mass) x 100*) of *Sterechinus neumayeri* from animals collected in April, May and August in 2017, and January and February in 2018. Error bars represent + standard error. N.D represents months where no data were available. **
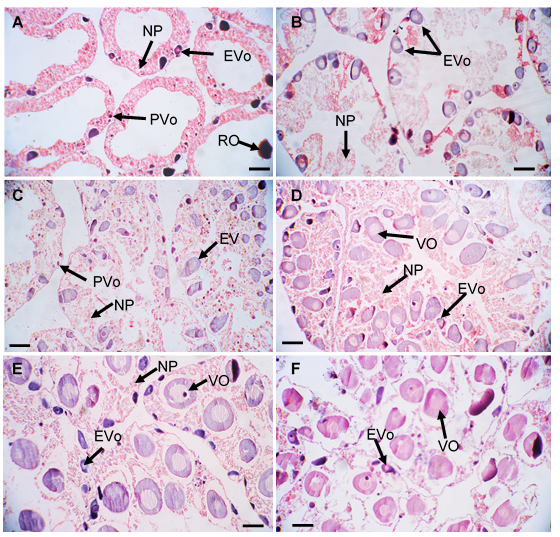
**

Figure S6: Histology sections of female *Sterechinus neumayeri* gonad tissue. A: Spent gonads with few pre-vitellogenic (PVo) and early-vitellogenic oocytes (EVo) beginning to appear around the edges of the gonad wall along with a lining of nutritive phagocytes (NP) and reabsorbing oocytes (RO) (November); B: Developing early-vitellogenic oocytes and thickening lining of nutritive phagocytes (February); C: Two cohorts of oocytes are present within the gonad, pre-vitellogenic and early-vitellogenic oocytes (March); D: Large, vitellogenic oocytes (VO) visible along with early-vitellogenic oocytes and further increase in nutritive phagocytes (April); E: Cohort of mature vitellogenic oocytes have reached maximal size and are ready to be released. Second cohort of early-vitellogenic oocytes present around the edges of the gonad wall (September); F: Mature vitellogenic oocytes and early-vitellogenic oocytes present in the gonad, reduction in nutrient phagocytes and increase in empty space as mature oocytes are spawned (October). Scale bars represent 100 μm.


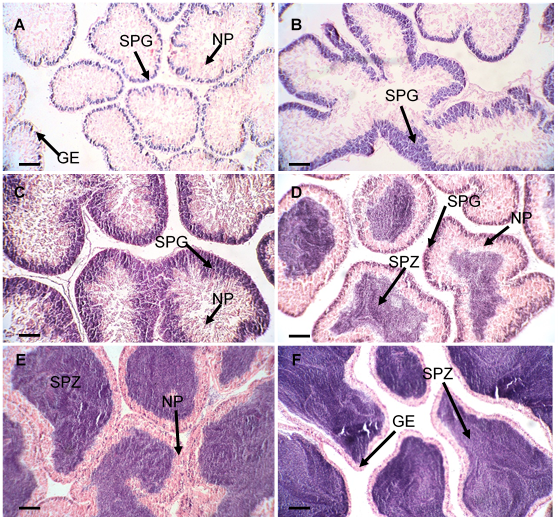
 Figure S7: Histology sections of male *Sterechinus neumayeri* gonad tissue showing different stages of maturation. A: Spawned/recovering gonads with mainly nutritive phagocytes (NP) with a thin layer of spermatogonia (SPG) around the edge of the germinal epithelium (GE) (February); B: Thickening of spermatogonia layer (March); C: Further thickening of spermatogonia layer and nutritive phagocytes (NP) (April); D: First signs of mature spermatozoa (SPZ) in the central lumen (May); E: Increased production of spermatozoa in central lumen, surrounded by a thick layer of nutritive phagocytes and absence of maturing spermatogonia (September); F: Final stages of maturity with mature spermatozoa present in large volumes in the central lumen with only a thin layer of nutritive phagocytes around the wall of the germinal epithelium (October). Scale bars represent 100 μm.


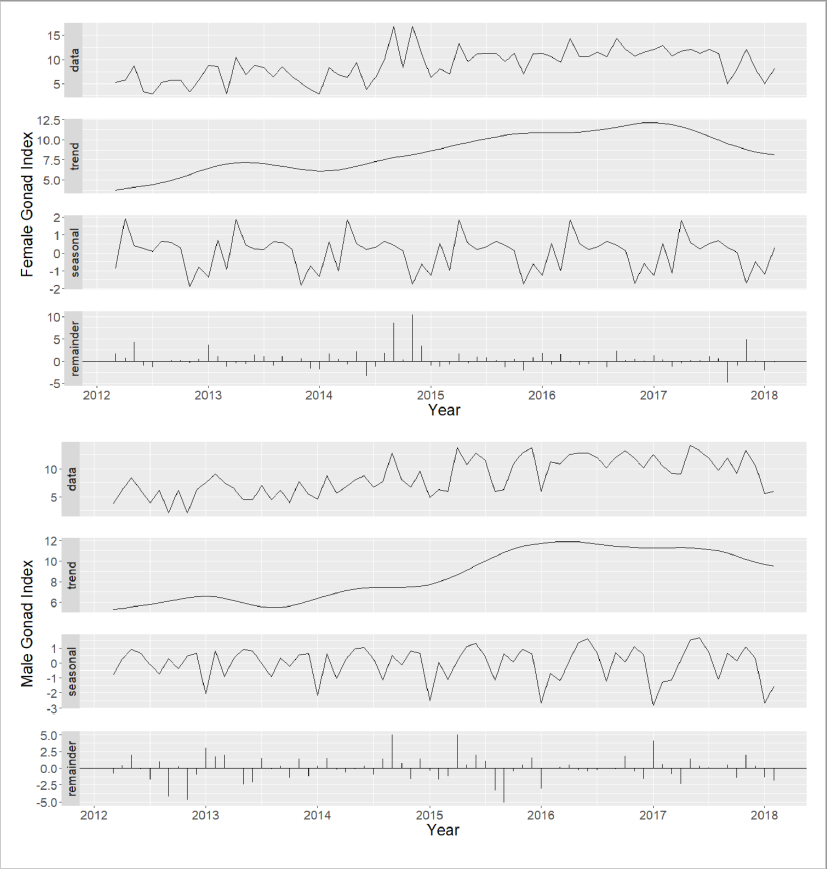
Figure S8: Decomposition analysis of female and male gonad index time-series, decomposed to the overall trend, seasonal cycle and remainder.

Table S1: Chi-squared analysis of sex ratio averaged within years and also overall from March 2012 - March 2018. F = Females, M = Males. p-values <0.05 are marked with an asterisk (*) to show significant differences.

| **Year** | **F** | **M** | **Chi-squared test**  **statistic** | **p-value** |
| --- | --- | --- | --- | --- |
| 2012 | 53 | 32 | 5.188 | 0.023* |
| 2013 | 73 | 87 | 1.225 | 0.268 |
| 2014 | 70 | 58 | 1.125 | 0.289 |
| 2015 | 64 | 53 | 1.034 | 0.309 |
| 2016 | 66 | 47 | 3.195 | 0.074 |
| 2017 | 51 | 31 | 4.878 | 0.027* |
| 2018 | 17 | 16 | 0.030 | 0.862 |
| Total | 408 | 310 | 13.376 | 0.000* |

Table S2: T-test analysis of sex difference for variables of animal size: test diameter and wet weight, and gonad index. Mean values for males and females are shown under columns ‘F’ (female mean) and ‘M’ (male mean). p-values <0.05 are marked with an asterisk (*) to show significant differences.

| **Variable** | **F** | **M** | **p-value** |
| --- | --- | --- | --- |
| Test diameter (mm) | 31.4 | 31.5 | 0.590 |
| Wet weight (g) | 12.9 | 13.3 | 0.323 |
| Gonad Index | 8.90 | 8.19 | 0.024* |

Table S3: ANOVA results for comparisons of oocyte Equivalent Circular Diameter between years for each month. June is exempt from the analysis as data were only collected in 2015. p-values < 0.05 were considered significant, F = F-statistic, df: degrees of freedom. Post-hoc Tukey pair-wise comparisons are listed where significant differences in the ANOVA were found with corresponding p value in adjacent column.

|  | **January** | **February** | **March** | | **April** | | **May** | | **July** | |
| --- | --- | --- | --- | --- | --- | --- | --- | --- | --- | --- |
|  | p = 0.251  F = 1.452  df = 2, 28 | p = 0.218  F = 1.522  df = 4, 34 | p < 0.001  F = 19.32  df = 5, 40 | | p = 0.005  F = 5.541  df = 3, 22 | | p < 0.001  F = 24.65  df = 3, 34 | | p = 0.008  F = 5.146  df = 3, 21 | |
| Tukey pair-  wise test |  |  | 2012 vs 2015  2016  2017 | p < 0.001  p < 0.001  p = 0.001 |  |  | 2012 vs 2013  2014  2015 | p < 0.001  p < 0.001  p = 0.001 | 2012 vs 2015 | p = 0.005 |
|  |  |  | 2013 vs 2015  2016  2017 | p < 0.001  p = 0.002  p = 0.007 | 2013 vs 2015  2016 | p = 0.009  p = 0.040 | 2013 vs 2014  2015 | p = 0.003  p = 0.019 |  |  |
|  |  |  | 2014 vs 2015  2016  2017 | p < 0.001  p < 0.001  p = 0.002 |  |  |  |  |  |  |
|  | **August** | | **September** | **October** | **November** | | **December** | |  | |
|  | p < 0.001  F = 18.29  df = 2, 18 | | p = 0.609  F = 0.275  df = 1, 13 | p = 0.940  F = 0.006  df = 1, 11 | p = 0.004  F = 7.865  df = 2, 17 | | p = 0.037  F = 5.758  df = 1, 10 | |  |  |
| Tukey pair-wise test |  |  |  |  | 2012 vs 2016 | p = 0.032 |  |  |  | |
|  | 2013 vs 2014  2016 | p < 0.001  p < 0.001 |  |  | 2013 vs 2016 | p = 0.003 | 2013 vs 2014 | p = 0.037 |  |  |

Table S4: Model selection process including the variables of weighted importance, followed by a ranking of automated model generation. The models are ranked according to the lowest Akaike Information Criterion (AIC). (+) = Terms included in model, AICc = Akaike Information Criterion corrected for small sample size, BIC = Bayesian Information Criterion, R^2^ = proportion of variance explained, d = difference in AICc relative to the top candidate model, w = Akaike weight.

| **Weighted importance** | |  | |  | |  | |  | |  | |
| --- | --- | --- | --- | --- | --- | --- | --- | --- | --- | --- | --- |
| **Time** | 0.999 |  |  | |  | |  | |  | |  |
| **Chlorophyll** | 0.999 |  |  | |  | |  | |  | |  |
| **SOI** | 0.955 |  |  | |  | |  | |  | |  |
| **SAM** | 0.398 |  |  | |  | |  | |  | |  |
| **Sex** | 0.822 |  |  | |  | |  | |  | |  |
|  | **Model 1** | **Model 2** | **Model 3** | | **Model 4** | | **Model 5** | | **Model 6** | |  |
| **Time** |  | + | + | |  | | + | | + | |  |
| **Chlorophyll** |  |  | + | |  | | + | | + | |  |
| **SOI** |  |  | + | |  | |  | | + | |  |
| **SAM** | + | + |  | |  | |  | | + | |  |
| **Sex** | + | + | + | | + | | + | | + | |  |
| **Time by Sex** | + |  |  | | + | |  | |  | |  |
| **Chlorophyll by Sex** | + | + |  | | + | |  | |  | |  |
| **SOI by Sex** | + | + |  | | + | | + | |  | |  |
| **SAM by Sex** |  |  | + | | + | | + | |  | |  |
| **AICc** | 1259.2 | 1263.7 | 1264.4 | | 1264.5 | | 1265.4 | | 1266.0 | |  |
| **BIC** | 1372.9 | 1353.6 | 1340.8 | | 1376.8 | | 1349.3 | | 1333.3 | |  |
| **R^2^** | 0.414 | 0.403 | 0.397 | | 0.412 | | 0.399 | | 0.392 | |  |
| **d** | 0.000 | 2.647 | 3.349 | | 3.420 | | 4.326 | | 4.941 | |  |
| **w** | 0.470 | 0.125 | 0.088 | | 0.085 | | | 0.054 | | 0.040 | |

Text S1: Decomposition analysis R code

#packages required

library(seasonal)

library(forecast)

library(magrittr)

library(mice)

library(tidyverse)

library(tidyr)

#gonad index for females

data <- gi_f

# OR gonad index for males

data <- gi_m

#average gi replicates by month

data_summary <- data %>%

group_by(date) %>%

summarize(mean = mean(gi))

#insert NAs in gaps

data <- data_summary %>% complete(date = seq.Date(min(date), max(date), by="month"))

#Need time series with no gaps, so fill missing months with predicted data using PMM (Predictive Mean Matching) <https://stefvanbuuren.name/fimd/sec-pmm.html>

[R Packages | Impute Missing Values In R (analyticsvidhya.com)](https://www.analyticsvidhya.com/blog/2016/03/tutorial-powerful-packages-imputing-missing-values/)

imputed_Data <- mice(data, m=5, maxit = 50, method = 'pmm')

summary(imputed_Data)

completeData <- complete(imputed_Data,2)

gidata <- subset(completeData, select = mean)# Selects collumns from the aggregated data into a new dataframe

#create a time series object

timeseries_gi <- ts(gidata, start = c(2012, 3), end =c(2018, #2), frequency = 12)

ts <- window(timeseries_gi, start=c(2012, 3), end=c(2018, 2))

plot(as.ts(ts))

#decomposition of data using X11 method. Based on classical decomposition, but includes many extra steps and features in order to overcome the drawbacks of classical decomposition.

fit <- seas(ts, x11 = "")

autoplot(fit)
